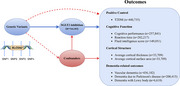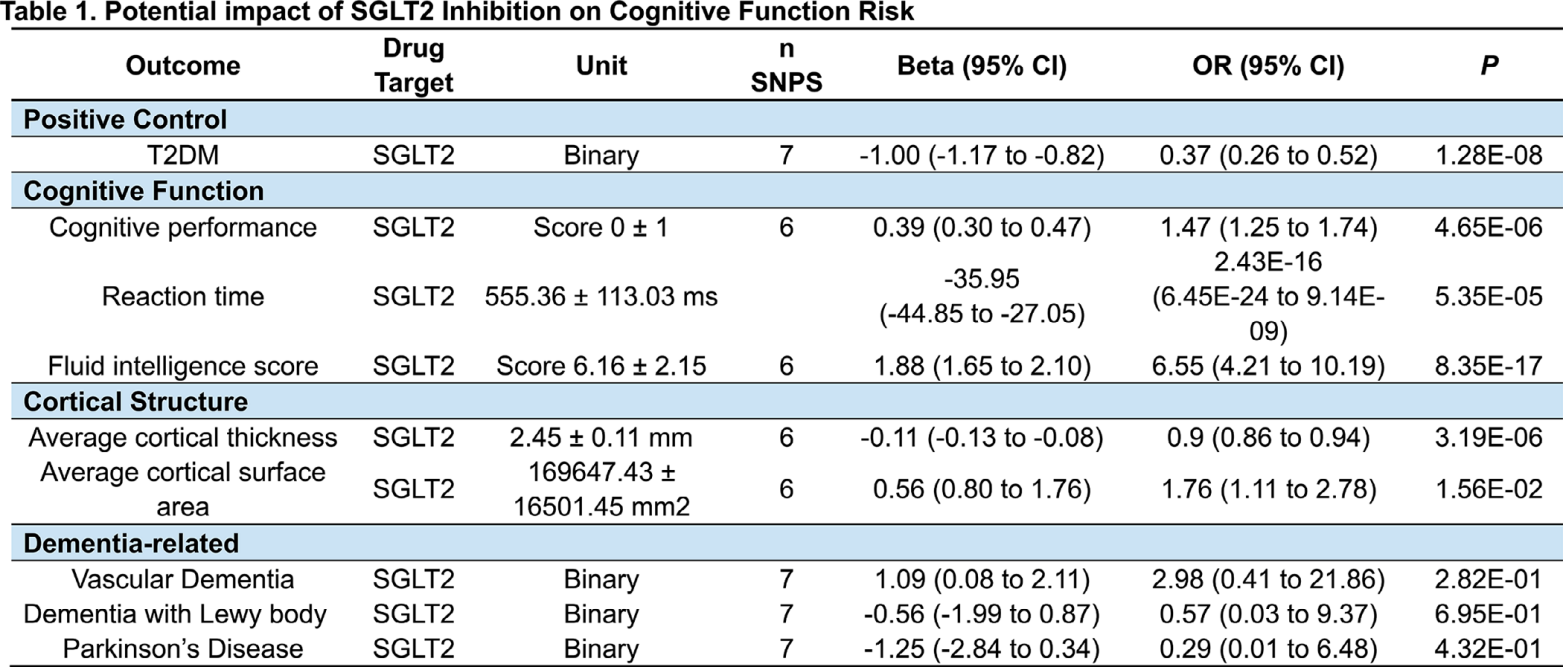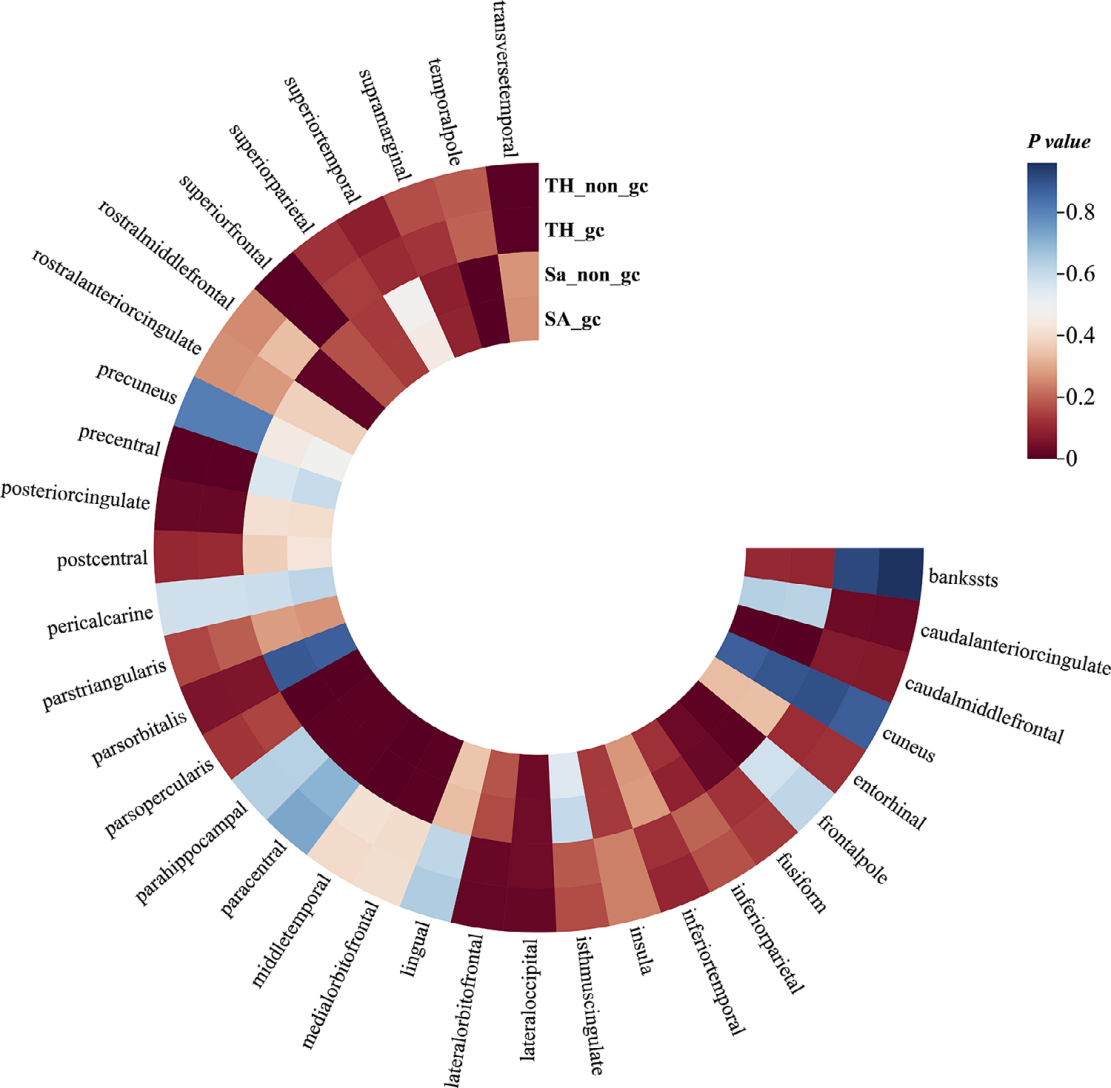# Mendelian Randomization Study on SGLT2 Inhibition and Cognitive Function

**DOI:** 10.1002/alz70859_106963

**Published:** 2025-12-26

**Authors:** Yuye Ning, Hao Yang, Guoliang Li

**Affiliations:** ^1^ Fuwai Hospital, Beijing, Beijing China; ^2^ Xuanwu hospital Capital Medical University, Beijing, Beijing China; ^3^ the First Affiliated Hospital of Xi'an Jiaotong University, Xi'an, Shaanxi China

## Abstract

**Background:**

Sodium–glucose cotransporter 2 inhibitors (SGLT2i) are commonly used in glucose‐lowering therapy and have demonstrated significant cardiovascular benefits. However, the long‐term effects of SGLT2i on neurocognitive function and brain structure remain unclear.

**Method:**

We extracted single nucleotide polymorphisms (SNPs) of SLC5A2, the gene encoding SGLT2, from genome‐wide association study (GWAS) summary statistics, predominantly based on European populations. Mendelian randomization analysis was conducted to establish a genetic instrument for SGLT2 inhibition, utilizing its association with SLC5A2 expression and reduced glycated hemoglobin levels. The effects of SGLT2 inhibition on brain structure and cognitive function were assessed using multiple cognition‐related outcomes to evaluate neurocognitive performance.

**Result:**

Our findings show that SGLT2 inhibition significantly reduces the risk of type 2 diabetes (β = ‐1.00, 95% CI: ‐1.17 to ‐0.82, OR = 0.37, 95% CI: 0.26 to 0.52, P = 1.28E‐08), confirming the efficacy of our genetic approach. In terms of cognitive function, SGLT2 inhibition was associated with improved cognitive performance (β = 0.39, 95% CI: 0.30 to 0.47, OR = 1.47, 95% CI: 1.25 to 1.74, P = 4.65E‐06), higher fluid intelligence scores (β = 1.88, 95% CI: 1.65 to 2.10, OR = 6.55, 95% CI: 4.21 to 10.19, P = 8.35E‐17), and shorter reaction times (β = ‐35.95, 95% CI: ‐44.85 to ‐27.05, P = 5.35E‐05). However, SGLT2 inhibition was linked to structural changes in the brain, including reduced mean cortical thickness (β = ‐0.11, 95% CI: ‐0.13 to ‐0.08, OR = 0.9, 95% CI: 0.86 to 0.94, P = 3.19E‐06) and increased cortical surface area (β = 0.56, 95% CI: 0.80 to 1.76, OR = 1.76, 95% CI: 1.11 to 2.78, P = 1.56E‐02). No significant associations were found between SGLT2 inhibition and dementia‐related outcomes, including vascular dementia (OR = 2.98, 95% CI: 0.41 to 21.86, P = 2.82E‐01), Lewy body dementia (OR = 0.57, 95% CI: 0.03 to 9.37, P = 6.95E‐01), and Parkinson's disease (OR = 0.29, 95% CI: 0.01 to 6.48, P = 4.32E‐01).

**Conclusion:**

SGLT2 inhibition may enhance cognitive function but could lead to structural changes in the brain, with no clear impact on dementia risk.